# The Nature of the Soul: Some Primitive Ideas

**Published:** 1916-07-01

**Authors:** 


### THE NATURE OF THE SOUL.

### Some Primitive Ideas.

In an interesting paper recently contributed to the Royal Society of Medicine Dr. Dan McKenzie discusses the folk-lore of rings and constrictions used as popular methods of treating diseases, and he endeavours to show that in some directions at least this practice was based on the view that it tended to prevent the escape of the soul. No doubt one notion underlying crude methods of constriction was that by its means undue growth could be checked. Hence the practice still prevailing in many countries of encircling the neck, in cases of goitre, with thread, or a strip of silk. Again, the use of rings as prophylactic or therapeutic agents is probably associated with a belief in the virtue of charms and has a basis in magic or even a semi-religious sanction. The shop-windows of to-day still display metal rings which are offered as cures for rheumatism, and on the same footing are rings boasting a protective influence and handed down in families as valuable heirlooms. But even when the checking of growth by compression and the mystic value of charms have been fully recognised there remain certain practices in which constriction is the prominent feature and for the explanation of which no obvious suggestion lies on the surface. The most prominent of these is the custom of tying a constricting band tightly round one or more of the limbs with the intention of checking not local but remote hæmorrhage, and it is here that Dr. McKenzie advances his suggestion that such a custom had as its primitive basis a belief that the method in question was necessary to hinder the escape of the soul from the body. The practice is still seen in Scotland where it is common to tie a thread round the thumb in bleeding from the nose, and similar methods are in vogue in many countries in the popular treatment of various hæmorrhages. Even in ancient and classical medical treatises the value of the ligature in the circumstances here mentioned is recognised, and such events as epistaxis, hæmoptysis, and menorrhagia are met by the application of ligatures round the arms and legs. How is the thesis supported that this practice in early days rested on an endeavour to retain the soul in the body? It is an attempt to answer this question that Dr. McKenzie examines some of the primitive ideas which have been held on the nature of the soul.

Quite early in human history was the association of the word "soul" with the idea of a breath or spirit introduced into the body from without. Thus in the Hebrew account of the Creation it is written that the Creator "formed man of the dust of the ground, and breathed into his nostrils the breath of life; and man became a living soul." But with such a supposition was widely associated the notion that the soul had a definite material existence—faint and shadowy no doubt, but still at times visible, at least to the penetrating observer, and even reproducing the physical outlines of the body from which at death, and possibly on other occasions, it escaped. In mediæval prints may be seen in the representation of dying persons a small figure emerging from the mouth, and there are savage tribes where an endeavour is made to hinder death by obstruction of the mouth and nostrils. On a similar basis rests the view that sleep means the temporary absence of the soul from the body, and a kindred notion extends to states of unconsciousness, such as syncope, epilepsy, coma, and the rest. Treated artistically this notion of a ghostly inhabitant on which the life and full activity of the body depend takes the form of some winged creature —a bird, a bee, a moth, or a butterfly. A step further leads to the recognition of such creatures as free or escaped human souls, and they have been "seen" to emerge from the waves on the sinking of a ship. Pigeons and doves take a prominent position in this association, and to this source may be traced a custom of applying pigeons to the feet of a person believed to be dying, the notion being presumably to restore or replace the soul which threatened to escape. Again, as an indication of the belief in the material nature of the soul is the view that the death agony is prolonged by the struggle of the soul to leave the body, and similarly that the final peace is only possible when by opening the window of the death chamber an exit is provided. In short, in popular creed, in art, in poetry, and in philosophy is displayed a belief in this ghostly inhabitant of the body, the flight of which is one aspect of the event we term death.

The next step is to connect the escape of the soul with loss of blood. Such a notion may have arisen in several ways. As severe hæmorrhage produced death, and as death meant the escape of the soul, an easy conclusion was that the soul was contained in the blood. Or again, a severe wound would not only cause bleeding, but it might be regarded as an opening through which the soul would take an opportunity to pass. In any such event there would be a natural effort to stop the bleeding, and this might be spoken of as a method of hindering the threatened exit of the soul. Keep in the blood and you will keep in the soul was possibly a prehistoric lesson in first-aid. Once grant such a position and a ligature round the extremities in the hope of stopping bleeding from the nose or other part is not difficult to explain. Such a ligature will, at all events, retain some blood in the body, and if it does this it will help to retain the soul, and consequently the life of the patient. Such in a summarised and somewhat abrupt form, is Dr. McKenzie's reasoned conclusion that the practice which he discusses has its origin in the notion that constriction of the limbs would prevent the escape of the soul. He has a special right to attention on any question of folk-lore, and even those whom he fails to convince will admire alike the range of his knowledge and his ingenuity in the application of it. His paper is full of interest and information.

